# The Local Option Act

**Published:** 1885-11

**Authors:** 


### THE LOCAL OPTION ACT.

The local option Act passed by the General Assembly of Georgia at its recent session, to provide "for preventing the evils of intemperance," by a vote of the population of each county, has become a law of the land by the sanction of the Governor on September 18, 1885.

By section 6th it is enacted: "That if a majority of the votes cast at any election, as by this Act provided, shall be against the sale, it shall not be lawful for any person, within the limits of such county, to sell or barter, for valuable consideration, either directly or indirectly, or give away to induce trade, at any place of business, or furnish at other public places any alcoholic, spirituous, malt or intoxicating liquors or intoxicating bitters, or other drinks, which if drank to excess will produce intoxication, under penalties hereinafter prescribed."

By section 8th it is further enacted: "That nothing in this Act shall be so construed as to prevent the manufacture, sale and use of domestic wines or cider, or the sale of wines for sacramental purposes; provided such wines or cider shall not be sold in bar-rooms by retail, nor shall anything herein contained prevent licensed druggists from selling or furnishing pure alcohol for medicinal, art, scientific and mechanical purposes."

The object of this bill is undoubtedly to promote the best interests of the men, women and children of our State, and in so far as it is calculated to suppress the vice of drunkenness must meet the approval of every good citizen of Georgia. But it becomes us to consider whether, in seeking to accomplish this most desirable result, there are incidental features connected with its execution, which may put in jeopardy the welfare of those for whose good it has been enacted. It behooves the medical profession to investigate the probable influence of a literal and faithful compliance with this Act upon the therapeutic resources of the physician in the treatment of disease, and to deliberate upon the measures which may be thought proper for correcting the injurious effects growing out of such law. If it is evident that all the favorable ends of wise legislation upon this subject may be secured without prejudice to the employment of various alcoholic prepartions as curative agents by the practitioners of medicine, most assuredly the law should be so construed or so modified as not to preclude the use of wine or beer, brandy or whisky, as medicines, when prescribed for the sick.

The great majority of medical men throughout the world concur in the conviction of the urgent necessity of all of these articles in certain conditions of the human system, and if their application is limited to the cure of disease, no possible detriment can occur to mankind. As our legislators could not be presumed to understand the importance of a prompt supply of alcoholic stimulants under circumstances of sudden prostration in sickness, or to avert the depresssing effects of slow, wasting disease, we must place the charitable construction upon this bill, that they looked solely to the prevention of the evils growing out of the abuse of intoxicating liquors by people in health. In this point of view, it would have been well to go even further in this measure of prohibition, so as to include the use of the preparations of opium, and of chloral; and to prevent the sale of these articles, even by druggists, without a physician's prescription. We are prepared to go even further than the enactment of a law against the making, buying, selling or giving of spirituous liquors for people in health; and we would punish the use of them, as well as the use of other narcotics, except as medicines. But this conviction of the direful consequences of such abuses of these agents should not and cannot militate against the proper employment of malt liquors, wine, brandy or whisky for medication, any more than it does against the judicious application of morphine or chloral, in the treatment of diseases. The conclusion that there is nothing intended to preclude the druggists from keeping these articles upon his shelves, among the other preparations required by the sick, and subject to the prescription of physicians, is perhaps a legitimate inference from the intended scope of this legislation "for preventing the evils of intemperance." This impression is strengthened by the special clause allowing druggists to sell alcohol, not only for medical purposes, but for other objects connected with art, science and mechanics. The general rule that holds in all legislation, that what is not included specifically in any prohibitory enactment is allowed, and the whole spirit of this Act is directed clearly against the ordinary indulgence of the appetite for intoxicating drinks, so that it is not by any means a forced construction of the law to conclude that it has no application to the sale of alcoholic liquors as medicines. But in case such a view is not legitimate, then it devolves upon the medical profession of Georgia to let its voice be heard in the interests of humanity, and appeal at once for such modification of the law as will authorize apothecaries to dispense these articles, under the prescriptions of physicians, for the relief of the sick. The penalty for any evasion of the true intent of this provision may be made so heavy, and under such conditions, that no one can be supplied with intoxicating drinks as a beverage, and we would advocate the most stringent regulations to guard against such abuses. But it is not requisite that the sick shall suffer for proper medication, because of the liability of people in health to make an improper use of intoxicating drinks, and it is a reflection upon the entire medical profession of this State, and upon the whole class of apothecaries doing a prescription business, to suppose that any harm can result from retaining alcoholic preparations on the list of the materia medica.

No portion of the community are more thoroughly convinced of the damning effects of intemperance than practitioners of medicine, and yet, believing, as they do, that all should abstain in health, they are profoundly and conscientiously impressed with the necessity of malt liquors, wine, brandy and whisky, in cases that no other medication can reach, and hence claim the right of prescribing these agents in like manner as other medicines.

---

### ITEMS.

DR. J. E. COOK, long a resident of Culloden, has removed to Barnesville.

DR. RICHARD MCSHERRY, one of the most prominent physicians of Baltimore, died October 7th, aged 68 years.

DR. HENRY F. CAMPBELL, of Augusta, has declined the vice-presidency of the International Medical Congress.

DR. JOHN L. ATLEE, Sr., one of the most distinguished of American physicians, and a founder and an ex-president of the American Medical Association, died at his home in Lancaster, Pa., on October 1st.

DR. WM. F. HOLT, of Macon, ex-president of the Medical Association of Georgia, and one of the most prominent physicians in the State, has gone to New York, where he will spend the winter in attending the clinics of the various colleges.

THE ATLANTA MEDICAL AND SURGICAL JOURNAL and *Daniel's Texas Medical Journal* to one address for one year for $3.50; regular price $4.50.

WE have received the programme of the proceedings of the second annual meeting of the New York State Medical Association to be held November 17, 18 and 19, 1885, at the Murray Hill Hotel, New York city.

THE medical colleges of this city have opened for the winter with a good attendance, possibly better than for several years past. The introductory lecture at the Atlanta College was delivered by Dr. J. D. Wilson, professor of chemistry. Dr. A. G. Hobbs, professor of diseases of the eye, ear and throat, delivered the introductory lecture at the Southern College.

THE RUMFORD CHEMICAL WORKS, Providence, R. I., manufacturers of Professor Horsford's Acid Phosphate, have recently purchased a commodious building and warehouse near their present location, where they propose to move their business a few months hence. This purchase has been necessitated by the demands of their large and increasing business, and it is pleasant to record such an evidence of well deserved success and prosperity.

REQUESTS TO OCULISTS.—*Mr. Editor:* Having taken charge of reporting for the "Revue Générale d'Ophthalmologic," edited by Dr. E. Meyer of Paris, and Dr. Dor of Lyon, on the progress of ophthalmology in our country, I beg leave to request all authors and publishers of ophthalmic works and papers to send me copies or reprints of their respective publications in order to enable me to give the most complete review of the current ophthalmic literature of our country in a periodical of the largest circulation among our profession. (Medical papers please copy.)

DR. M. LANDESBERG.

40 W. 34th Street, New York, October 3, 1885.

WE had a pleasant call a few days ago from Dr. C. W. Nutting, of Etna, California. Dr. Nutting was at one time demonstrator of anatomy in the Atlanta Medical College. He resigned this place in 1878 and went to California, where he has been engaged in the practice of medicine ever since. He is here on a visit to his father, and will spend several weeks in the city. We are pleased to state that he has been successful in his practice and has accumulated quite a fortune.

A GERMAN OPINION OF THE INTERNATIONAL CONGRESS.—The success of the International Medical Congress is questionable. The American Medical Association has deposed the original committee and tried to substitute a new one. We hope that this domestic quarrel of the American physicians will soon be amicably adjusted, for European ophthalmologists will hardly feel inclined to take part in the meeting of a section from which Knapp and Agnew have been excluded.—*Centralblatt f. pract. Augenheilkunde, Berlin.*

TWO FRAUDS.—T. H. Bryant, of Waukesha, Wis., and E. S. Morgan, of Keyport, N. J., are both frauds, and we would advise our readers to keep clear of them. Of Bryant, the *Texas Medical Journal* has this to say: "And now comes T. H. Bryant, 'proprietor' 'Waukesha Glen," the 'Queen of Mineral Waters,' and poses as the prince of advertising frauds! He is liberal with his ads., five pages at a pop, but does not pay a cent. Beat us out of $26.66, the price of five pages one insertion, and we consider ourselves lucky to have been warned by two other leading journals worse stuck. He trades on wind as well as water. *This is free.*"

A MERITED COMPLIMENT.—One of the wisest selections of presidents of the different sections of the Medical Congress was made when Dr. A. W. Calhoun, of this city, was appointed president of the section of Ophthalmology. There is not a man in this country who ranks Dr. C. in his profession, either practically or theoretically. A few days since the question was asked, who, of all men of this city and section, could we the least afford to lose? The answer came promptly from numbers, Dr. A. W. Calhoun. If all the selections made were as fitting as Dr. Calhoun, the congress of 1887 will be the most brilliant the medical profession has ever held.—*Southern Dental Journal.*

EXPLOSIVE PHYSIC.—A list has just been published in the *Union Pharmaceutique* of accidents which have recently occurred during the preparation or carriage of explosive substances used in medicine. At Strassburg, a chemist's assistant was changing some lycopodium-powder from one bottle to another ; the particles that escaped mixed with the air, a jet of gas was burning, and a slight explosion occurred. The frightened assistant dropped the jar containing the lycopodium, the room was at once filled with the powder, and a violent explosion took place. Chlorate and permanganate of potash are also dangerous. M. Meyet has stated that a tooth-powder composed of chlorate of potash and cachou has been known to explode in the mouth of a person engaged in brushing his teeth. A druggist who dried some hypophosphite of lime in a receptacle containing sand was killed by its explosion. Oxalate and citrate of lime are also explosive, but only at a high temperature. Pills of permanganate of potash have been known to explode spontaneously. A mixture of perchloride of iron and glycerine exploded in the pocket of a patient who carried it. An eminent chemist at Paris prepared ozone with powders composed of equal parts of peroxide of manganese, permanganate of potassium, and pulverized oxalic acid. He took every recognised precaution, and the mixture was corked up in a bottle ; a few minutes afterwards, an explosion took place, and the bottle was reduced to atoms.—*British Medical Journal.*

DR. WM. H. COGGESHALL, of Richmond, one of the editors of the *Virginia Medical Monthly*, is dead.

ISAAC PHILLIPS, at 13 N. Broad street, has a large stock of dissecting cases, to which the attention of medical students is directed.

D. R. Bogue, of Montreal, is canvassing the physicians in Georgia in the interest cf the *Medical and Surgical Directory* of the United States, published by R. L. Polk & Co., of Detroit.

NATROLITHIC WATER.—Attention is directed to the advertisement of this water in this issue of THE JOURNAL. See the advertisement and write for samples, which will be supplied to physicians.

AN old Highlander, rather fond of his toddy, was ordered by his physician not to exceed one ounce of spirits daily. The old gentleman was rather dubious about the amount, and asked his son, a schoolboy, how much an ounce was. "Sixteen drams," was the reply. "What a guid doctor," exclaimed the Highlander; "run and tell Donald McTavish and Big John tae cam doon the nicht."—*Birmingham Medical Review*.

METEOROLOGICAL SUMMARY FOR ATLANTA FOR SEPTEMBER, 1885.

| | |
|---|---|
| Highest Temperature, September 15th, | 87.2 |
| Lowest Temperature, September 24th, | 53.0 |
| Greatest Daily Range of Temperature, | 19.0 |
| Least Daily Range of Temperature, | 2.5 |
| Mean Daily Range of Temperature, | 12 6 |
| Mean Daily Dew-point, | 62.6 |
| Mean Daily Relative Humidity, | 81.1 |
| Prevailing Direction of Wind, | East. |
| Total Movement of Wind, | 7,086 Miles. |
| Highest Velocity of Wind and Direction, | 28 Miles, E. |
| Number of Foggy Days, | None. |
| Number of Clear Days, | 8 |
| Number of Fair Days, | 13 |
| Number of Cloudy Days, | 9 |
| Number of Days on which Rain or Snow fell, | 12 |

